# Supplementary material for: Key factors in supporting adolescents to achieve high self-esteem and a positive body image: A qualitative community-based study
Source: PLoS One. 2025 Feb 25;20(2):e0318989. doi: 10.1371/journal.pone.0318989 (PMC11856398; doi:10.1371/journal.pone.0318989)
Supplement: S2 File — (DOCX) [file pone.0318989.s002.docx]

Supplementary File 2: Appendix 2_ Generic interview script

(Adolescents, parents and teachers)

**1. Cordial greeting and introduction of the interviewer.**

**2. Delimit the reason for the interview and its duration:**

- Inform that the questions are designed to provide information about activities and possible intervention strategies to improve self-esteem and positive body image in school-aged adolescents.

- Inform that this is an interview that will be recorded as indicated in the informed consent and will last approximately 30-45 minutes.

**3. Interview:**

- Could you please tell us your age (all profiles), academic course (adolescents and teachers), university degree (teachers) or level of education (parents), family relationship, number of children and their ages (parents)?

- What are your views on self-esteem and how it can affect self-image?

- What factors do you think make it difficult for adolescents to achieve high self-esteem and a positive body image?

- What factors do you think might facilitate adolescents in achieving high self-esteem and a positive body image?

- What kinds of activities do you think could help adolescents improve their self-esteem and positive body image?

- How do you think these activities or interventions should be designed?

- In which settings do you think these activities would be most effective?

- Who do you think should be involved in these activities and how?

- What issues do you think should be addressed in future activities?

**4. Cordial closure and thanks:**

- Are there any aspects we haven’t covered that you would like to add?
