# Supplementary material for: Key factors in supporting adolescents to achieve high self-esteem and a positive body image: A qualitative community-based study
Source: PLoS One. 2025 Feb 25;20(2):e0318989. doi: 10.1371/journal.pone.0318989 (PMC11856398; doi:10.1371/journal.pone.0318989)
Supplement: S3 File — (DOCX) [file pone.0318989.s003.docx]

**S3 Table 2. Statements by teachers about examples of classroom activities.**

| **Subtheme** | **Quotations** |
| --- | --- |
| Lack of training | *‘We did an activity called “Great”, and it involved saying positive things, not telling lies but saying positive things about yourself, teachers, classmates..., well, activities of this kind’. T4* |
|  | *‘Group activities, that's mainly it, examples, some short films, a movie; I had already done this, with situations and scenarios, and then they discuss, they debate, and then also share. Something else I have done is to assign roles, and then they have to say a positive quality about each student, always positive things, never negative. So, when students receive all the positive comments from their classmates about them, they feel really good and at the same time, it's unexpected for them’. T7* |
|  | *‘The other day, I conducted an activity based on these comments that were made, and I used images of girls... those that boys follow and like, and then I showed them the opposite, images of boys that girls follow, right? So, I made them stand up in class and said... guys, are you just like these guys who appear on social media? No? Well, don't demand that the girls be the same as them, right? It was an exercise for them to see that one thing is the reality of everyday life, and the other is what they want to appear like when there are retouched images (...) From here, they can discuss together in small groups how to address this and how to make the other person feel better’. T3* |
|  | *‘One activity we carried out was: they paired up facing each other, right? And what they had to do was draw each other, and once they were done, show each other the drawing and explain why they had done it that way or why they had highlighted a specific aspect, right? (...) We also did the “Delegates 3D” project as part of emotional education. So, what they had to do was create a project to help in the classroom, their group, in the space (...) They would then use different dynamics to create, well, what project they want to do... how and who they want to help... and how they want to do it’. T2* |
