# Supplementary material for: Key factors in supporting adolescents to achieve high self-esteem and a positive body image: A qualitative community-based study
Source: PLoS One. 2025 Feb 25;20(2):e0318989. doi: 10.1371/journal.pone.0318989 (PMC11856398; doi:10.1371/journal.pone.0318989)
Supplement: Attachment — Submitted filename: Review_comments.docx [file pone.0318989.s004.docx]

**Review**

**Abstracts**

The abstract effectively outlines with the aim of the study, methods and key finding of the study. It ends with a relevant conclusion that indicates the social and public impacts.

**Recommendations:**

1. **Introduction:** As there is no direct analytical proof such as regression analysis, significance (p-value), statement like this in line 92- **Body dissatisfaction significantly affects adolescents' mental health, leading to issues with body weight control, low self-esteem, and poor school performance**. This statement could be softened by rephrasing such as “Body dissatisfaction appears to affect/ can be affect”, in order to avoid overgeneralization.
2. **Methods:** Specifying the number and demographics of participants upfront like “A total of 24 interviews with adolescents, parents, and teachers in Spain were conducted for this study” would be clearer for the readers.
3. **Conclusion:** Suggest adding implication for broader public health policies to enhance the abstract’s impact.

**Introduction**

Introduction of this study provides a thorough background of body image (BI) and self-esteem (SE) issues among adolescents, linking those concern to societal influences like social media (SM).

**Recommendations:**

1. **Indicate the study gap:** Clearly articulate the gap in prior studies to increase impact of this study. As an example, statement like this can be used “Existing studies focus primarily on individual factors but lack insights into community-based interventions targeting SE and BI.".
2. **Structure:** Concluding the introduction with a specific research question or hypothesis would be helpful to set expectations for the readers.

**Methods**

Well-detailed, with clear steps for recruitment, data collection, and analysis.

**Recommendations:**

1. **Recruitment and participants:** As line 190 “Purposive sampling” used in recruitment, elaborate that and address potential biases in using peer nomination.
2. **Data analysis:** In line 237- “Inductive and deductive coding were performed to identify the meaning units of the text.” Explain how inter-coder reliability was ensured during thematic analysis. If it is possible provide an overview of the coding process, including an example of how themes were derived from raw data.

**Results**

This section is comprehensive and effective uses of participants quotes to illustrates key finding were seen.

**Recommendations:**

1. **Theme Presentation:** Introduce a summary table with the main themes, subthemes, and representative quotes for quick reference.
2. **Quotes:** Shorten participant quotes to highlight only the most impactful insights.

**Discussion**

Discussion section demonstrated a strong connection to the literature and emphasizes actionable interventions.

**Recommendations:**

1. **Policy Implications:** Include examples of how findings can inform school-based policies or public health initiatives.
2. **Limitations:** Expend on how the rural Catalonian setting may limit generalizability and suggest strategies for urban applications.

**Conclusion**

It summarized findings and give importance to multi-level interventions involving families, schools, and communities.

**Recommendation:** Conclusion could’ve been end with a stronger call to action for researchers, educators, and policymakers.

**Abbreviations:** In line 139 abbreviation like PBI was used but full meaning of the abbreviation was not used prior of that line. Ensure consistency, use full form once then use abbreviation thereafter.
